# When Eating Becomes Torturous: Understanding Nutrition-Related Cancer Treatment Side Effects among Individuals with Cancer and Their Caregivers

**DOI:** 10.3390/nu14020356

**Published:** 2022-01-14

**Authors:** Brandy-Joe Milliron, Lora Packel, Dan Dychtwald, Cynthia Klobodu, Laura Pontiggia, Ochi Ogbogu, Byron Barksdale, Jonathan Deutsch

**Affiliations:** 1 Department of Nutrition Sciences, College of Nursing and Health Professions, Drexel University, 1601 Cherry Street, Philadelphia, PA 19102, USA; ddychtwald@ifh.rutgers.edu (D.D.); cnk48@drexel.edu (C.K.); 2 Department of Food and Hospitality Management, College of Nursing and Health Professions, Drexel University, 3141 Chestnut Street, Philadelphia, PA 19104, USA; st96d633@drexel.edu; 3 Department of Physical Therapy, University of the Sciences, 600 S. 43rd Street, Philadelphia, PA 19104, USA; l.packel@usciences.edu; 4 Institute of Emerging Health Professions, Thomas Jefferson University, 901 Walnut Street, Philadelphia, PA 19107, USA; laura.pontiggia@jefferson.edu; 5 AstraZeneca Hope Lodge of the American Cancer Society, 110 W Laurel Ave., Cheltenham, PA 19012, USA; ochi.ogbogu@cancer.org (O.O.); byron.barksdale@cancer.org (B.B.)

**Keywords:** side effects, nutrition, survivorship, eating-related distress, food, caregiver

## Abstract

Individuals living with cancer often experience multiple nutrition-related side effects from cancer treatment, including changes in taste and smell, nausea, diarrhea, loss of appetite, and pain during eating. These side effects can profoundly impact nutritional status and quality of life. The purpose of this study was to explore experiences with nutrition-related cancer treatment side effects among cancer patients and their family caregivers, the way they manage such side effects, and the resulting changes in food preferences and behaviors. Structured surveys and in-depth interviews were conducted. Interviews focused on the presence and management of treatment side effects, how those changes influenced food preferences, and the extent to which they interfered with quality of life. Most patients (72%) reported treatment side effects; 61% reported that these side effects impacted their eating and drinking. Common side effects included fatigue (58%), dry mouth (30%), nausea (24%), constipation (20%) and diarrhea (20%). Six overarching qualitative themes were identified: Spiral of side effects; Pain of eating; Burden of eating; Loss of taste/change in taste; Symptom management; and Solutions. The authors conclude with implications for food and nutrition practice—moving beyond traditional recommendations of what to eat or avoid—to consider the overall patient and caregiver experience.

## 1. Introduction

Cancer treatments, especially chemotherapy, can cause sudden and dramatic changes in taste and smell, nausea and vomiting, diarrhea, loss of appetite, sores in the mouth and throat, trouble swallowing, dry mouth, and fatigue [1]. The likelihood that a person with cancer will experience at least one treatment-induced side effect is high. In a large survey of patients undergoing chemotherapy or radiotherapy for cancer in the United States (US), 88% of the respondents reported at least one side effect [2]. In another US study, over 67% of patients undergoing treatment reported at least one taste or smell alteration, and approximately 40% of patients reported a decreased appetite since beginning treatment [3]. The likelihood of experiencing a cluster of side effects is also high. Among the 86% of patient participants in an Australian prospective cohort study who reported at least one side effect, 67% experienced six or more side effects with over one-third reporting a grade IV side effect (indicative of life-threatening consequences and where urgent intervention is recommended) [4,5].

The role that caregivers play in helping to mitigate the nutrition and dietary side effects of cancer treatment is critically important, yet often overlooked. Moreover, the impact on the dietary intake and health of a person taking care of someone with nutritional side effects is understudied. Defined as individuals who care for relatives or friends diagnosed with cancer as a primary diagnosis, cancer caregivers are instrumental in providing vital care. In addition to emotional, informational, and functional support, caregivers often provide skilled care, including activities that range from dressing wounds, cleaning ports, and managing treatment side effects. The roles and responsibilities of a cancer caregiver begin at diagnosis, and vary with type, stage and severity of cancer, and can last for months or several years until recovery or loss of life of the person being cared for.

While research has documented the frequency and severity of the side effects experienced by patients related to cancer treatment, we know little about dyadic interactions when trying to manage (and cope with) treatment side effects. Therefore, the purpose of this study was to explore patients’ and caregivers’ experiences with cancer treatment side effects, including the way they manage those side effects and the resulting changes in food preferences and behaviors. These insights of the lived experiences of families navigating cancer treatment provide additional guidance on how to tailor supportive care interventions to mitigate treatment side effects and improve eating-related distress for both patients and their families. This study is a component of a broader program of research to guide the development of tailored nutrition and physical activity programming to improve cancer survivorship and caregiver health.

## 2. Materials and Methods

### 2.1. Study Design

This multiple methods study employed a quantitative structured survey and semi-structured qualitative in-depth interviews using an inductive thematic approach. The investigation was nested within a larger, convergent mixed methods study that aimed to inform the development of tailored nutrition and physical activity programming to improve cancer survivorship and caregiver health.

### 2.2. Participants

Individuals living with cancer who were undergoing cancer treatment (patients) and their caregivers were recruited through a partnership with The American Cancer Society’s AstraZeneca Hope Lodge (Hope Lodge) in Cheltenham, PA, which provides housing for individuals receiving outpatient cancer treatment more than 40 miles away from their homes. All guests must be accompanied by an adult caregiver during their stay. Patients at Hope Lodge who were undergoing active treatment, and their caregivers, were recruited through flyers, word of mouth, and announcements at community dinners. Participants received a maximum of $50 for their participation in study activities. Patients were eligible to participate if they were 18 years of age or older, going through active cancer treatment, and had the ability to read and speak English. Caregivers were eligible if they were 18 years of age or older, the caregiver of someone going through active cancer treatment, and had the ability to read and speak English. This study was approved by the Institutional Review Boards at Drexel University and University of the Sciences; and written consent was obtained from all individuals prior to participation. Data were collected between June 2017 and September 2019.

### 2.3. Quantitative Data Collection and Analysis

A structured survey was used to obtain the participant’s demographic and personal characteristics, including cancer and caregiving characteristics. For patients, seven questions were asked about treatment side effects and their resulting food preferences and aversions, which were adapted from a previously developed self-report questionnaire [6]. Specifically, these questions addressed the occurrence of side effects; whether side effects disturbed eating and drinking; ability to cook; dietary behaviors; food aversions; food attributes that stimulate appetite; and food preferences. These questions were closed-ended, offered options to provide alternative answers, and participants could choose more than one answer when applicable.

Descriptive characteristics from demographics and the prevalence and severity of nutrition-related treatment side effects in patients were summarized using means and standard deviations for quantitative variables and frequencies and percentages for categorical variables. Quantitative statistical analyses were performed using SAS v. 9.4 (SAS Institute, Inc., Cary, NC, USA).

### 2.4. Qualitative Data Collection and Analysis

In-depth interviews were conducted with patients and caregivers separately. All interviews were semi-structured, and open-ended questions were followed by probes and transitions. In-depth interviews focused on the presence and management of nutrition-related treatment side effects, such as altered taste and flavor, and how those changes influenced food preferences and the extent to which they interfered with participant quality of life. Interviews with caregivers also asked about caregivers’ own nutrition goals and how the cancer experience (such as treatment side effects) impacted their diet. Separate interview guides were created for patients and caregivers. Interviews lasted approximately 30–45 min and were conducted in a private room.

Interviews were audio recorded and transcribed verbatim. Transcripts were first read multiple times by five study team members. Transcripts were analyzed using an inductive thematic analysis approach. Following the method outlined by Ely et al. (1997), data representing each code were printed and three coders manually manipulated the data into piles, over multiple rounds, to tell a collective story representing the lived experience of the patients. These collections of data were then reviewed by the other researchers in the study to assess if they rang true to the overall impression of the setting and the experiences of the participants. To ensure respect for each participant, the words of the participants are presented without or with or minimal editing to allow the reader to understand the participant experience and assess for themselves whether the themes from the analysis ring true to the words of the participants.

## 3. Results

### 3.1. Sample Characteristics

One hundred and two participants (50 patients; 52 caregivers) completed the structured survey and 40 participants (20 patients; 20 caregivers) completed in-depth interviews. Forty-two percent of patients and nearly 81% of caregivers were female (Table 1). The majority of both groups were older than 55 years of age. For patients, just over 80% identified as Caucasian, 19% as African American, and a little over 6% identified as Hispanic or Latinx. For caregivers, 88% identified as Caucasian, 4% as African American, and nearly 6% identified as Hispanic or Latinx. The participants were highly educated; 62% of patients and just over 67% of caregivers reported educational level of college or greater.

All of the patients were undergoing active cancer treatment. A variety of cancer types were presented in the sample; hematological cancers (20.8%), head, neck, or lung cancer (20.8%), gastrointestinal cancers (16.7%), or other solid tumors (41.7%). Twenty-five percent of patients reported 10 or more days of absence due to treatment, and 12% of caregivers reported 10 or more days of absence due to their caregiving role. Caregivers reported providing a median of five hours per day of caregiving responsibilities, seven days per week.

### 3.2. Treatment Side Effects, Important Food Attributes and Preferences 

Seventy-two percent of patients experienced treatment side effects, and of those, 61% reported that the side effects they experienced had an impact on what they were able to eat and drink (Table 2). The most common side effects included fatigue (58%), dry mouth (30%), nausea (24%), constipation (20%) and diarrhea (20%). Other side effects included swallowing and chewing difficulties, mouth ulcers, vomiting, hypersensitivity to odors, and persistent foreign taste. Only 28% of patients felt confident that they would be able to cook as soon as they returned home.

**Figure 1:**
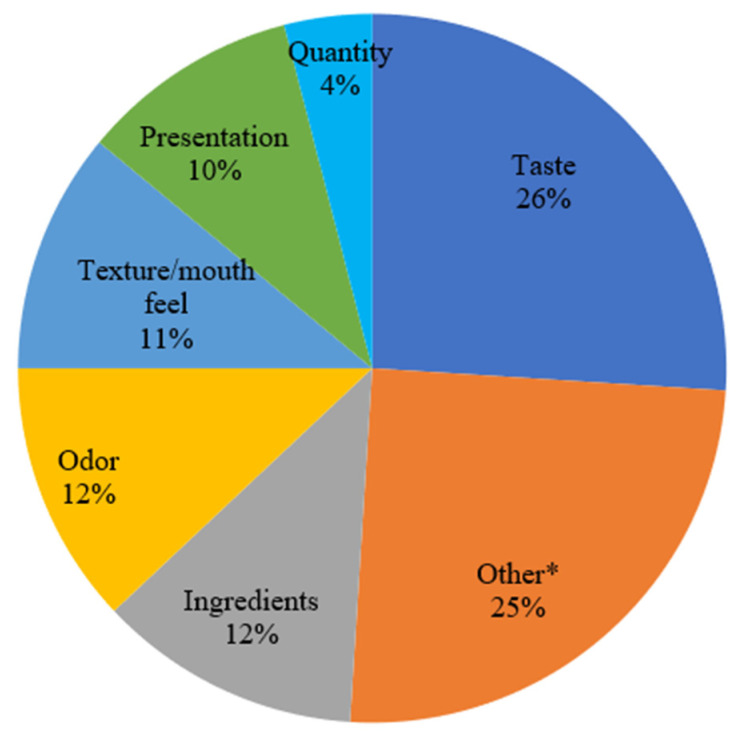
Patient ranking of important sensory qualities that stimulate appetite. * Other: Patients reported the following “Other” important sensory qualities to stimulate appetite: calm environment, atmosphere, time of day, healthfulness, and colors.

Patients also ranked a list of the most important characteristics for stimulating appetite. On a seven-point scale, patients were asked to rank the following characteristics in descending order: taste, ingredients, odor, consistency or texture, quantity, presentation, and other. Taste was the most frequently cited characteristic to stimulate appetite (median score of 6), followed by ingredients (median score 3.5), odor (4), consistency/texture (4), quantity (2), and presentation (4; Figure 1). While not all patients ranked “other” qualities as important attributes, those who did ranked their importance as very high (median score of 6). Patients mentioned calm environment, atmosphere, time of day, healthfulness, and colors as “other” important attributes.

**Figure 2:**
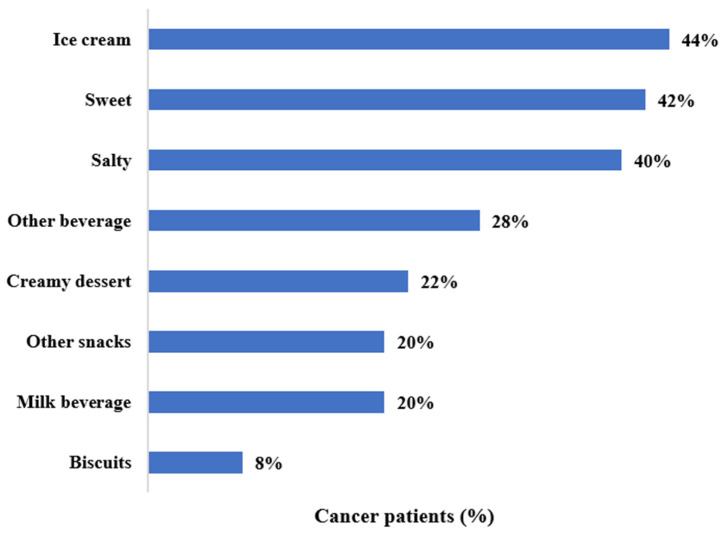
Preferred types of snacks. Distribution of most frequently reported foods or flavors that patients preferred in the preceding month among individuals with cancer.

Regarding snack preferences in the past month, the most frequently reported foods or flavors that patients preferred were ice cream (44%), sweet (42%) and salty snacks (40%; Figure 2). Less frequently, patients reported preferring “other beverages” (such as water and Gatorade; 28%), creamy desserts (22%), milk beverages (20%), “other snacks” (such as fruits and vegetables; breads and pasta; yogurt; nuts and dried fruit), and biscuits (8%).

### 3.3. Qualitative Findings 

We identified six themes from the analysis: (1) Spiral of side effects; (2) Pain of eating; (3) Burden of eating; (4) Loss of taste/change in taste; (5) Symptom management; and (6) Solutions. We also identified three through-lines, which served as a second layer of themes that appeared throughout our participants’ experiences. These through-lines included: (A) Caregiver empathy and sharing experience of treatment; (B) Complementary and alternative medicine in conflict with traditional oncology protocols; (C) Be warned, but you’re on your own to figure this out. Table 3 and Table 4 summarize the themes and through-lines using exemplar quotes. Table S1 displays the characteristics of a sample of the participants we interviewed.

#### 3.3.1. Theme 1: Spiral of Side Effects

One of the themes that emerged from the qualitative interviews is that, contrary to what a healthcare professional or pharmacist might tell a patient about a drug, its intended treatment, and its potential side effects, the lived experience of a patient and their caregiver is not so orderly and linear. Appetite, nausea, diarrhea and constipation, and other digestive functions are embedded in a complex nexus of treatments and symptoms that overlap. Patients take drugs to address the nutrition related treatment side effects, which in turn necessitates coping with, or treating with, an additional drug, which leads to further side effects.

*“Ah, the thing is, I take so many pills that I don’t, that everything…you get one pill for something and then they give you something else and they give you a pill for that…and it’s just never ending. So, I never know which one is giving what so that’s, that’s the only thing that I research a lot ah, possible side effects.”—Damien*

Alfred confirms,

*“I mean everything is about strength of side effects, then you take something for that side effect and then, you know, you get another side effect from that and…”*

The patient message resonates with the caregivers as well.

*“Oh, yes, the vomiting, nauseousness [sic], loss of hair, fatigue. Of course you’re gonna have fatigue with cancer, and yeah. We expected them. I went through it three years ago, so I kinda knew what he was gonna do, and he did too, because he was my caregiver, so we were kind of blessed that way (laughs), you know?”—Lenora*

*“He has side effects…Mostly cramps, fatigue, some pain—he don’t sleep very well. He’s depressed…I think my role is to support him…Try to help him hang on.”—Douglas*

#### 3.3.2. Theme 2: Pain of Eating

Eating is one of the great pleasures of life. However, for a patient or caregiver, the blessing of a good meal can become overshadowed by the pain of trying to get—and keep—a meal down. This is not just to say that it is physically taxing for patients, but it is also difficult for caregivers to watch and try to remedy. From the patient perspective, John shares his experience.

*“So, when I swallow, it was like forcing a ball in my mouth you know, and like ripping my throat. And I became leery about how I would swallow and what I would swallow. Um…and the tear begins to keep going on and I took pain pills.”*

Additionally, later…

*“You know, they said it was like guaranteed that I would like go through these symptoms, especially since I am being treated for my tongue too, back part of my tongue. The ah, ah then it would make ah, ah large difference you know in my eating and my swallowing you know, and it came out to be true.”*

Caregivers empathize with the pain of their loved one.

*“His taste has completely left, he has no taste. Um, extremely dry mouth. A lot of pain swallowing, the skin is starting to turn colors. He has lost a lot of weight I’d say. He started out at 210, he’s 197 now. And the doctor is like ‘No! Beef him up and…’ Let’s see what else… Irritable (laughs). Oh—and he’s got the ulcers, he has the open ulcers in the mouth. They warned us… but it’s nothing like actually going through it.”—Janice*

#### 3.3.3. Theme 3: Burden of Eating

Related to the theme ‘Pain of eating’, is food and nutrition being a burden more broadly, for patients and caregivers. In the following quotations and stories, Douglas, a caregiver, points out that both he and his son have both lost their appetite.

*“We don’t have normal appetite because he doesn’t.”—Douglas*

Roy shares his struggle after he received a stem cell transplant; and Janice, a caregiver, expresses feelings of defeat and worry.

*“The first time I really ever felt any effect, physiologically from treatments or my cancer or anything, was the stem cell transplant I had a year ago. And that…they told me going in that they said it would be it would be it would be a rough experience for me, you know, to get through there. I can remember uh…losing my appetite and…which, which was…I mean I like food…so losing my appetite was something I didn’t cherish at all. And food just sort of it just lost its appeal you know from a nutrition standpoint.”—Roy*

*“I’m starting to feel defeated. Because I’m like if you lose any more weight, you know, and I’m so afraid for this, it’s like—he is doing the best he can. He’s eating. He is eating. He’s just not eating as much. He went from eating a whole bowl of oatmeal, to a half a bowl, to now it’s a couple of bites. I’ll just drink an Ensure and I’ll be alright and I’m watching this. And in his mind, he’s eating a lot, he’s doing great. And I’m like “Really?” (laughs)…you’re losing weight. I’m worried. I’m starting to worry. I just don’t know what else to do.”—Janice*

#### 3.3.4. Theme 4: Loss of Taste/Change in Taste

The burden of appetite does not exist in a vacuum but is tied inextricably to loss or change in taste. Because taste is both personal and subjective (how am I to know how something tastes to you?) and challenging to measure (we can measure changes to your bloodwork or urine but not to your palate), this is a poorly understood side effect that does not garner as much attention or empathy from healthcare professionals as it should. However, to a patient, it can be exhausting.

*“Well the thing they did not they did not…they did not clue me on ahead of time was that the low dose radiation would wipe out my taste buds. For, for a significant period of time, probably, a good month or 6 weeks. That’s tough! It’s tough to lose your ability to taste food. You know, I mean pile that on top of questionable appetite you know…That—I wasn’t expecting and that was probably, through all of this, I’d have to say, through all the fatigue, fatigue and everything I can handle it, but the inability to taste food properly, and then when the buds came back. It came back in waves like salt, you know so everything tasted salty you know, or bitter, or lack of sweet sense, and so you’d eat something that’s supposed to be nice and sweet and yummy and you know it’s just ‘psst’. I’ll tell you it’s a really, really, really tough time.—Roy*

*“I would try to eat something different at every day just to see if something ah, made sense, if something changed. Ah, there was this different taste I could catch from something in the…I tried, I tried, but it didn’t happen. Nothing would taste…like anything.”—Damien*

For the caregiver, it can feel helpless and futile to get their partner to eat.

*“It started out with everything tasted like metal for a couple weeks. And now, since last week, he has zero taste (laughs). Unless it’s super strong, like if he eats something that has citric acid, he’ll taste a twinge of it or if it’s super spicy. Other than that, he has zero taste. So, I try my best—‘You got to try to imagine it, try to imagine it… Eat with your eyes.’ But that doesn’t work, of course. So I just say—‘Just let it go down. When your stomach feels full, stop.’ (Laughs). But it’s like—trying to get him to want to eat. And he says ‘Well what’s the point? I can’t taste it.’ And I say ‘Because you have to eat.’ So, because he doesn’t even want to eat now, that’s the problem I’m dealing with now. But now, they’re threatening him with-- if you lose any more weight, you’re going to get a feeding tube. So now it’s like ‘Give me potatoes!’ (laughs).”—Janice*

#### 3.3.5. Theme 5: Symptom Management

Related to the challenges of eating and the spiral of side effects is a parallel spiral of treatments, solutions, and suggestions being proposed by healthcare professionals, caregivers and the patients themselves to attempt to ameliorate the symptoms. This puts additional work and burden on patients and caregivers, especially when they are not effective, resulting in further frustration. Damien, a patient, shares his experience following a (common) suggestion that patients hear, which is to eat spicy or overly flavorful food…

*“They just recommended that kids like Doritos because they have a strong taste, so that, that (laughs) was like the only thing that kids would eat cause it, ah, ah…I don’t know, it has a strong taste so that, that would make them taste a little bit, but ah…There was, there was nothing you could do about it, about not having taste buds…”*

   *Interviewer: “Did you try the Doritos?”*

    *“I think I did (laughs)…”*

      *Interviewer: “Could you taste them?”*

       *“No.”*

Michael, a caregiver, shares their dissatisfaction with medication management for symptom alleviation.

*“Yeah, they offered some—you know, the first thing is—well, first off, if I back up, she had some constipation, because of the pain they did give her a little oxycodone, very small amounts, which constipated her. So, they gave her Colace. So, their basic thing is to drug it and then the diarrhea, they were saying the Imodium. But we did talk to the naturopath there who we really respect and she really respects, I mean, cause she—they talk their own lingo, so they’re—so the naturopath has been very helpful because her thing is food and things.”*

#### 3.3.6. Theme 6: Solutions

With all of these side effects, and their fraught causes and treatments, both patients and caregivers grasp for solutions, especially from complementary and alternative medicine and looking towards “super foods”, that can somehow be more powerful in nutrition and healing than their inherent properties allow.

*“For the bone, she was taking (stutters) some joint support, some (pause) different herbs and things like that and it’s a whole—it’s about 30 some herbs and stuff, but she was taking a lot of that. She was taking some stuff to build up the blood. She was taking some stuff to, like—guava and some other things that would help attack the cancer. So she was doing a lot of herbal stuff to actually attack and treat the cancer that was non- “medicinal” and this was prior to getting (stutters) the herbs.”—Michael*

*“I’m thinking the bone broth and then protein shakes rather than that Ensure refrigerator. [Laughs] Yeah. We’re both not into the Ensure, that’s for sure. Although she was because part of her problem before she was diagnosed was she was having trouble eating. So, she was eating—she was downing Slimfast shakes because she was forcing herself to eat something. And you know, I’m like, let me make these shakes for you. They’re good, you know. It’s plant-based protein and coconut oil, fruits and greens. So, she has had those with me and she is open to that. I said, ‘Before you drink anything like that other stuff, please let me just do this with you.’ [Laughs]”—Beth*

## 4. Discussion

For most individuals living with cancer, the experience of nutrition-related side effects during cancer treatment is uniquely distressing for the individual and their family. In this study, the majority of patients (72%) reported treatment side effects and 61% reported that these side effects impacted their eating and drinking. The most common side effects included fatigue (58%), dry mouth (30%), nausea (24%), constipation (20%) and diarrhea (20%). In addition to the side effects prevalent among our patient population, people with cancer often experience changes in taste and smell, which include experiencing pervasive bad taste (in all food), being unable to perceive food taste, and experiencing modifications of the perceived taste of food [7]. These changes, in addition to treatment-related fatigue and the milieu of other side effects, are associated with a decline in nutritional status as they lead to decreases in appetite, avoidance of certain foods, and reduced food intake [8,9,10,11,12]. The problem does not stop there. The occurrence of treatment-induced side effects has also been associated with lower health-related quality of life [13], higher distress [14,15], impaired physical function [16], reduced tolerance to cancer treatment [17], and reduced survival [18]. Of particular concern, malnutrition is associated with an increased risk of frailty [19,20,21]. For individuals living with (and beyond) cancer, frailty has been associated with an increased risk for adverse events, incidence of chronic diseases, and an increased risk for overall mortality [22,23].

Although many caregivers attribute personal fulfillment to their caregiving role, cancer caregiving has also been associated with extreme physical, psychological, and financial distress, many reporting that caregiving is a driver of their worsened health status [24]. In fact, the compromised health status of chronically stressed (or distressed) caregivers can diminish their capacity to provide high quality care [25,26]. Among the most common sources of psychological distress for cancer caregivers is difficulty in managing the side effects of treatment. One study estimated that up to 50% of cancer caregivers report lacking adequate knowledge of how to manage nutrition-related cancer symptoms and treatment side effects, and more than a quarter lack confidence in the quality of care they provide at home [27].

The unmet needs of individuals with cancer and their caregivers described herein call for continued research and the development of programs tailored towards assisting cancer patients and their caregivers to navigate treatment-related side effects. Hence, the primary aim of this study was to (qualitatively) explore patients’ and caregivers’ experiences with nutrition-related cancer treatment side effects, including how they manage those side effects and the resulting changes in food preferences and behaviors. The following six themes emerged: (1) Spiral of side effects; (2) Pain of eating; (3) Burden of eating; (4) Loss of taste/change in taste; (5) Symptom management; and (6) Solutions, work both independently and together. Independently, they represent baskets or buckets of common experience that can provide guidance to patients, caregivers, and healthcare professionals regarding what might be expected in treatment. Together, they suggest some through-lines that make our understanding of the patient and caregiver experience more meaningful (Table 4 and Table 5). These are:

- Caregiver empathy and sharing experience of treatment
- Complementary and alternative medicine in conflict with traditional oncology protocols
- Be warned, but you’re on your own to figure this out

### 4.1. Through-Line A: Caregiver Empathy and Sharing Experience of Treatment

One of the things the research team observed in interviewing patients and caregivers, and in performing the subsequent transcription and analysis, is that it is sometimes challenging to differentiate between patient and caregiver. There is a practical reason that patients and caregivers became conflated—as treatment progressed, their empathy for one another deepened and there was a mutual sharing of the side effect experience and the burden. This burden becomes heavy as cancer care is often focused on treating the cancer, with less emphasis on the side effects, shifting a lot of the management responsibility to the care partners.

Patients and their caregivers experience a wide range of emotional and psychosocial consequences as a result of treatment side effects. When faced with the reduced ability to enjoy food and eating together, patients and caregivers share feelings of anger, anxiety, guilt, disappointment, loss and grief, shame and suffering [28,29,30]. While a patient’s emotional responses may be driven by the experience of the side effect (loss of taste, pain during eating), caregivers experience these same emotions because of their high sense of responsibility for the patient well-being [30]. Furthermore, food and eating serve a central role in people’s social and emotional lives, especially in their close relationships [31]. Food sharing and food offering facilitate the development and strengthening of social bonds, serving as catalysts for relational closeness and cooperation within families and between romantic partners [29,32,33,34].

Research has also shown that sharing food preferences, food provision and food preparation are behaviors that can regulate interpersonal relationships, whereas discrepancies in these food behaviors can lead to relational discord [35,36,37,38]. In the context of cancer treatment, food offering and food sharing cycles can be severely disrupted, as patients are too fatigued to participate in food provision and preparation, experience changes in their food preferences due to treatment side effects, or are unable to eat or taste food altogether.

In summary, caregivers share the psychosocial and emotional impacts of treatment side effects and are at high risk of negative physical, psychosocial and emotional consequences. They experience a high sense of responsibility in alleviating side effects and providing care more broadly. Therefore, caregivers should be monitored by healthcare professionals during patient care, which is a central tenet of person-centered care. Developing and testing interventions that improve caregivers’ capacity to provide high-quality care while simultaneously improving their nutrition and long-term health is a top priority. Additional research is needed to develop nutrition and culinary dyadal interventions that harness the influential role that each member of the dyad holds, while simultaneously being responsive to their unique needs.

### 4.2. Through-Line B: Complementary and Alternative Medicine in Conflict with Traditional Oncology Protocols

As mentioned above, evidence-based oncology protocols are used for the treatment of cancer. However, when it comes to the treatment of side effects, the tent is much bigger. Complementary and alternative medicine (CAM), herbalism, functional ingredients and folk medicine are fair game for trying to stimulate appetite, suppress nausea, and soothe the pain of swallowing and so on. Often, caregivers and patients are left to source these treatments on their own—healthcare professionals may not have an awareness about such remedies or may even actively discourage their adoption, for fear of contraindicating a treatment. Table 4 explores some of these treatments and where they may conflict with the oncology protocol.

The use of CAM activities, which include medical and other health-related products and practices that are not a part of standard medical care, has increased in recent decades [39]. Findings from a systematic review and meta-analysis reported that the estimated global prevalence of CAM use among cancer patients was 40%; the highest use reported in the US [40]. A more recent analysis estimated the prevalence of CAM use by cancer type in their sample of individuals with breast (39.6%), prostate (44.4%), ovarian (37.0%), and colorectal (38.7%) cancer [41]. Reasons for CAM use vary, and include intentions such as improved physical well-being, alleviation of treatment side effects and long-term effects, and refraining from conventional treatment all together, among others [42]. The reported benefits of CAM are mixed. Many report that they are not sure, while others, including one caregiver in this study, report “less pain” and “better mobility” [42]. It is important to note there is a potential for both positive and negative effects of CAM during and after treatment.

Participants in our study expressed a lack of communication with their healthcare team related to CAM approaches, and this has also been reported by others [42]. Open communication among patients, caregivers and cancer care providers, inclusive of patient interest in and preferences in CAM, should be encouraged as a key component of person-centered care. A transition toward integrative oncology, or the use of evidence-based CAM strategies alongside conventional therapies, could also be explored and considered.

### 4.3. Through-Line C: Be Warned, but You’re on Your Own to Figure This Out

Healthcare professionals are skilled at listing potential side effects of a drug or treatment. Patients and caregivers report that healthcare providers are less skilled at guiding them through side effect management. The result is a sense that patients have been warned that these side effects are coming without having received adequate guidance or support to cope with them. Relatedly, they have been warned in brief about side effects such as nausea or change in taste perception. However, brief summaries do not explain just how grave and debilitating these side effects can be, in terms of, for example, a complete disgust and distaste for food of any sort. In this way, the term “side effect” tends to minimize this very real and substantial impact such treatments can have. Table 4 also illustrates this frustration expressed by both patients and caregivers.

Being ill-prepared for the impact of cancer treatment, even when forewarned by their cancer care team, is a re-occurring barrier documented in the literature. Despite the anticipation of treatment side effects, patients and caregivers report feeling unprepared for their severity [43]. Some families report receiving ineffective suggestions for dealing with changes in taste and smell, and in some cases, such suggestions exacerbated other side effects. For example, patients report they are often told to salt their food before eating it to accentuate flavors. Yet, the added salt creates thirst and dry mouth, and often does little to change the flavor of their food [44].

While further intervention research is needed to elucidate the most effective approaches for preventing and mitigating treatment side effects, there is also a real need for improved transparency and support. Providers should be transparent about anticipated side-effects, their severity and management. Sharing experiences of patients and caregivers, such as those illustrated in this paper, has the potential to both improve transparency and reassure that patients and families are not alone in their struggle.

## 5. Strengths and Limitations

The strengths of this study were the inclusion of both patient and caregiver perspectives and the timeframe of data collection. Participants completed study activities while undergoing cancer treatment. This allowed participants to describe their experiences in real-time or to reflect on their experiences shortly after they occurred. This study was not without its limitations. Participants were recruited using convenience sampling and the data collected in this study were cross-sectional and self-reported. Therefore, causal inferences cannot be made and some of our findings could be susceptible to social desirability bias, subjectivity and recall bias. Our sample was racially homogenous, older in age, and the majority of caregivers were women while the majority of patients were men; these findings may not be generalizable to other cancer patients and caregivers.

## 6. Conclusions

Eating is among our most pleasurable, inter-connected experiences. Cancer, its treatment, and its side effects quickly transform this positive and pleasurable experience into a curse—an obligation that must be “managed” and “handled” for both patients and caregivers, with little direction provided by the care team. Understanding the lived experience of both cancer patients and caregivers regarding nutrition-related cancer treatment side effects can yield multiple benefits. First, healthcare providers can become better versed in helping patients and caregivers to anticipate and prepare for treatment side effects. Second, the study revealed the lack of a linear and evidence-based approach to mitigate treatment side effects and this is something that should be further investigated. Better anticipating and managing these nutrition-related cancer treatment side effects can position both the patient and caregiver to better achieve improved nutrition and related health outcomes. There is a great need to identify effective interventions to reduce nutrition-related treatment side effects in people with cancer as well as mechanisms to increase caregiver preparedness and self-efficacy to manage side effects. Such interventions could dramatically improve the physical, emotional, and psychosocial health of patients and their caregivers.

## Figures and Tables

**Table 1:** Participant demographic and personal characteristics.

		Cancer Patients (*N* = 50)	Caregivers (*N* = 52)
Demographics			
Gender, n (%)			
	Female	21 (42.00%)	42 (80.77%)
	Male	29 (58.00%)	10 (19.23%)
Age Range, n (%)			
	18–54 years	9 (18.00%)	15 (28.85%)
	55+	41 (82.00%)	37 (71.15%)
Race, n (%)			
	African American	9 (19.15%)	2 (4.00%)
	Caucasian	38 (80.85%)	44 (88.00%)
	Other	0 (0%)	4 (8.00%)
Hispanic or Latino, n (%)			
	Yes	3 (6.38%)	3 (5.77%)
	No	44 (93.62%)	49 (94.23%)
Level of Education, n (%) (Grouped)			
	HS Graduate or less	12 (24.00%)	6 (11.54%)
	Some college	7 (14.00%)	11 (21.15%)
	College or greater	31 (62.00%)	35 (67.31%)
Adequate Financial Support, n (%)			
	Yes	40 (80.00%)	44 (84.62%)
	No	10 (20.00%)	8 (15.38%)
Personal Characteristics			
Cancer type, n (%)			
	GI cancer	8 (16.67%)	
	Head, Neck or Lung	10 (20.83%)	
	Hematologic Cancers	10 (20.83%)	
	Other Solid	20 (41.67%)	
Absence due to Trt or Role, n (%)			
	None or NA	34 (70.83%)	33 (66.00%)
	1–9 days	2 (4.17%)	11 (22.00%)
	10+ Days	12 (25.00%)	6 (12.00%)
Average number days per week caregiving			
	Median (IQR)		7 (5, 7)
	Range		0, 7
Averagenumber hours per day caregiving			
	Median (IQR)		5 (2.5, 10)
	Range		0, 24

**Table 2:** Side effects, important food attributes, and snack preferences reported by cancer patients undergoing treatment.

Side Effects Reported	Cancer Patients (*N* = 50) *
Fatigue	29 (58.00%)
Dry mouth	15 (30.00%)
Nausea	12 (24.00%)
Constipation	10 (20.00%)
Diarrhea	10 (20.00%)
Swallowing difficulties	8 (16.00%)
Mouth ulcers	8 (16.00%)
Persistent taste	7 (14.00%)
Other	6 (12.00%)
Chewing difficulties	4 (8.00%)
Hypersensitivity to odors	4 (8.00%)
Vomiting	2 (4.00%)
Interference/disturbance of eating and drinking due to side effects (If Yes to side-effect experienced question) (*N* = 36)	
Yes	22 (61.11%)
No	14 (38.89%)

* Data are displayed as n (%).

**Table 3:** Themes and Exemplar Quotes.

Themes	Examples
Spiral of side effects	“He has side effects…Mostly cramps, fatigue, some pain—he don’t sleep very well. He’s depressed…I think my role is to support him…Try to help him hang on.”—Douglas
	“Ah, the thing is, I take so many pills that I don’t, that everything…you get one pill for something and then they give you something else and they give you a pill for that…and it’s just never ending. So, I never know which one is giving what so that’s, that’s the only thing that I research a lot ah, possible side effects.”—Damien
	“I mean everything is about strength of side effects, then you take something for that side effect and then, you know, you get another side effect from that and…”—Alfred
	“Oh, yes, the vomiting, nauseousness, loss of hair, fatigue. Of course you’re gonna have fatigue with cancer, and yeah. We expected them. I went through it three years ago, so I kinda knew what he was gonna do, and he did too, because he was my caregiver, so we were kind of blessed that way (laughs), you know?”—Lenora
	So, they don’t actually tell you everything. Well, they weren’t surprised, but I think was and I would say, “what’s this” and they would say, “well, we did touch on that a little bit”. Well, if they touched on it, I was sleeping or (laughing)—my chemo didn’t hear it.—Susan
	“They told me all the side effects and they gave me a ton of pills and every time I had this effect, they took that pill; then, when I had that effect, they took that pill.”—Henry
Pain of eating	“So, when I swallow, it was like forcing a ball in my mouth you know, and like ripping my throat. And I became leery about how I would swallow and what I would swallow. Um…and the tare begins to keep going on and I took pain pills.”—John
	“His taste has completely left, he has no taste. Um, extremely dry mouth. A lot of pain swallowing, the skin is starting to turn colors. He has lost a lot of weight I’d say. He started out at 210, he’s 197 now. And the doctor is like “No! Beef him up and…” Let’s see what else…Irritable (laughs), quick.”
	“Oh—and he’s got the ulcers, he has the open ulcers in the mouth. They warned us…but it’s nothing like actually going through it.”—Janice
	“You know, they said it was like guaranteed that I would like go through these symptoms, especially since I am being treated for my tongue too, back part of my tongue. The ah, ah then it would make ah, ah large difference you know in my eating and my swallowing you know, and it came out to be true.”—John
	“The chemo gives him the cold. You can’t touch cold, so he has to drink room temp, and if not, he can feel it in his throat. I don’t know how to describe it. When I had it, it felt like you’re swallowing glass. His hasn’t come to that yet, but his hands and feet and that, when he touches cold, it affects him, and that’s from the chemo, and we’re expecting that.”—Lenora
Burden of eating	“The first time I really ever felt any effect, physiologically from treatments or my cancer or anything, was the stem cell transplant I had a year ago. And and that…they told me going in that they said it would be it would be it would be a rough experience for me, you know, to get through there. I can remember uh…losing my appetite and…which, which was…I mean I like food…so losing my appetite was something I didn’t cherish at all. And food just sort of it just lost its appeal you know from a nutrition standpoint.”—Roy
	“We don’t have normal appetite because he doesn’t (either).”—Douglas
	“I’m starting to feel defeated. Because I’m like if you lose any more weight, you know, and I’m so afraid for this, it’s like—he is doing the best he can. He’s eating. He is eating. He’s just not eating as much. He went from eating a whole bowl of oatmeal, to a half a bowl, to now it’s a couple of bites. I’ll just drink an Ensure and I’ll be alright and I’m watching this. And in his mind, he’s eating a lot, he’s doing great. And I’m like “Really?” (laughs)…you’re losing weight. I’m worried. I’m starting to worry. I just don’t know what else to do.”—Janice
Loss of taste/change in taste	“It started out with everything tasted like metal for a couple weeks. And now, since last week, he has zero taste (laughs). Unless it’s super strong, like if he eats something that has citric acid, he’ll taste a twinge of it or if it’s super spicy. Other than that, he has zero taste. So, I try my best—‘You got to try to imagine it, try to imagine it. Eat with your eyes. But that doesn’t work, of course. So I just say—‘Just let it go down. When your stomach feels full, stop.’ (Laughs). But it’s like—trying to get him to want to eat. And he says ‘Well what’s the point? I can’t taste it.’ And I say ‘Because you have to eat.’ So, because he doesn’t even want to eat now, that’s the problem I’m dealing with now. But now, they’re threatening him with—if you lose any more weight, you’re going to get a feeding tube. So now it’s like ‘Give me potatoes!’ (laughs).”—Janice
	“I would try to eat something different at every day just to see if something ah, made sense, if something changed. Ah, there was this different taste I could catch from something in the…I tried, I tried, but it didn’t happen. Nothing would taste…like anything.”—Damien
	“Well the thing they did not they did not…they did not clue me on ahead of time was that the low dose radiation would wipe out my taste buds. For, for a significant period of time, probably, a good month or 6 weeks. That’s tough! It’s tough to lose your ability to taste food. You know, I mean pile that on top of questionable appetite you know…That I wasn’t expecting and that was probably, through all of this, I’d have to say, through all the fatigue, fatigue and everything I can handle it, but the inability to taste food properly, and then when the buds came back. It came back in waves like salt, you know so everything tasted salty you know, or bitter, or lack of sweet sense, and so you’d eat something that’s supposed to be nice and sweet and yummy and you know it’s just ‘psst’. I’ll tell you it’s a really, really, really tough time.—Roy
Symptom management	“They just recommended that kids like Doritos because they have a strong taste, so that, that (laughs) was like the only thing that kids would eat cause it, ah, ah…I don’t know, it has a strong taste so that, that would make them taste a little bit, but ah…There was, there was nothing you could do about it, about not having taste buds…” Interviewer: “Did you try the Doritos?” “I think I did (laughs)…” Interviewer: “Could you taste them?” No.”—Damien
	“Yeah, they offered some—you know, the first thing is—well, first off, if I back up, she had some constipation, because of the pain they did give her a little oxycodone, very small amounts, which constipated her. So, they gave her Colace. So, their basic thing is to drug it and then the diarrhea, they were saying the Imodium. But we did talk to the naturopath there who we really respect and she really respects, I mean ’cause she—they talk their own lingo, so they’re—so the naturopath has been very helpful because her thing is food and things.”—Michael
	“So—and the truth is, as far as pain goes, when we got to the hospital in December, before—when we just—and the doctors kind of said—we didn’t meet the naturopath, that was the only one we didn’t meet, unfortunately, and they told her to stop all the herbs, she walked in, she couldn’t walk out. The pain had increased. When she started just saying screw it, I’m gonna start taking the herbs actually decreased her pain, and we’re talking like 8 to 10 down to like a 3 to 4 pain.”—Michael
	“The hair loss, I mean that really isn’t a problem. The fatigue that really hasn’t been a problem either because they suggested ways to break that so the main one we have been doing is, my wife and I have been walking maybe we would walk different trails or around the closest neighborhood, I would say about a half hour each day just to stay active and that’s really helped fight off the fatigue.”—Carter
	“But her pain is much more intense now in the evening and in the middle of the night and stuff, and it wakes her up and, you know, sometimes she is like screaming in pain and stuff like that. And so I think activity keeps you off of the pain. When you’re inactive and you’re not doing anything then you can dwell on it. And I also think you dwell on it, you know, in a negative way because it hurts, it hurts, it hurts, and you can’t distract yourself from it.”—Michael
Solutions	“For the bone, she was taking (stutters) some joint support, some (pause) different herbs and things like that and it’s a whole—it’s about 30 some herbs and stuff, but she was taking a lot of that. She was taking some stuff to build up the blood. She was taking some stuff to, like—guava and some other things that would help attack the cancer. So she was doing a lot of herbal stuff to actually attack and treat the cancer that was non- “medicinal” and this was prior to getting (stutters) the herbs.”—Michael
	“I just have to force it on him, and remind him. I tell him “it’s breakfast”—because we’re on a schedule—“It’s lunch, it’s dinner.” I became more forceful with him. Before I was—you do what you gotta do. But now I’m the one that’s more forceful. And, I try to make things a bit more seasoned, than I would normally do, because hopefully he’d be able to taste it (laughs). And then he’s throwing it out because he doesn’t want to eat it all. So now I’m starting to feel bad about that. Now you’re wasting food. I don’t know…I just keep asking for tips and hints on the internet, the doctors, somebody give me some tips. Because I don’t know what else to do, because he’s not eating.”—Janice
	“I’m thinking the bone broth and then protein shakes rather than that Ensure refrigerator. [Laughs] Yeah. We’re both not into the Ensure, that’s for sure. Although she was because part of her problem before she was diagnosed was she was having trouble eating. So, she was eating—she was downing Slimfast shakes because she was forcing herself to eat something. And you know, I’m like, let me make these shakes for you. They’re good, you know. It’s plant-based protein and coconut oil, fruits and greens. So, she has had those with me and she is open to that. I said, ‘Before you drink anything like that other stuff, please let me just do this with you.’ [Laughs]”—Beth

**Table 4:** Through-lines and Exemplar Quotations.

Through-Lines	Examples
Caregiver empathy and sharing experience of treatment	“I’m thinking the bone broth and then protein shakes rather than that Ensure refrigerator. [Laughs] Yeah. We’re both not into the Ensure, that’s for sure. Although she was because part of her problem before she was diagnosed was she was having trouble eating. So, she was eating—she was downing Slimfast shakes because she was forcing herself to eat something. And you know, I’m like, let me make these shakes for you. They’re good, you know. It’s plant-based protein and coconut oil, fruits and greens. So, she has had those with me and she is open to that. I said, ‘Before you drink anything like that other stuff, please let me just do this with you.’ [Laughs]”—Beth
	“I just have to force it on him, and remind him. I tell him “it’s breakfast”—because we’re on a schedule—“It’s lunch, it’s dinner.” I became more forceful with him. Before I was—you do what you gotta do. But now I’m the one that’s more forceful. And, I try to make things a bit more seasoned, than I would normally do, because hopefully he’d be able to taste it (laughs). And then he’s throwing it out because he doesn’t want to eat it all. So now I’m starting to feel bad about that. Now you’re wasting food. I don’t know…I just keep asking for tips and hints on the internet, the doctors, somebody give me some tips. Because I don’t know what else to do, because he’s not eating.”—Janice
	“But her pain is much more intense now in the evening and in the middle of the night and stuff, and it wakes her up and, you know, sometimes she is like screaming in pain and stuff like that. And so I think activity keeps you off of the pain. When you’re inactive and you’re not doing anything then you can dwell on it. And I also think you dwell on it, you know, in a negative way because it hurts, it hurts, it hurts, and you can’t distract yourself from it.”—Michael
	“The hair loss, I mean that really isn’t a problem. The fatigue that really hasn’t been a problem either because they suggested ways to break that so the main one we have been doing is, my wife and I have been walking maybe we would walk different trails or around the closest neighborhood, I would say about a half hour each day just to stay active and that’s really helped fight off the fatigue.”—Carter
	“Yeah, they offered some—you know, the first thing is—well, first off, if I back up, she had some constipation, because of the pain they did give her a little oxycodone, very small amounts, which constipated her. So, they gave her Colace. So, their basic thing is to drug it and then the diarrhea, they were saying the Imodium. But we did talk to the naturopath there who we really respect and she really respects, I mean ’cause she—they talk their own lingo, so they’re—so the naturopath has been very helpful because her thing is food and things.”—Michael
	“It started out with everything tasted like metal for a couple weeks. And now, since last week, he has zero taste (laughs). Unless it’s super strong, like if he eats something that has citric acid, he’ll taste a twinge of it or if it’s super spicy. Other than that, he has zero taste. So, I try my best—‘You got to try to imagine it, try to imagine it. Eat with your eyes.’ But that doesn’t work, of course. So I just say—‘Just let it go down. When your stomach feels full, stop.’ (Laughs). But it’s like—trying to get him to want to eat. And he says ‘Well what’s the point? I can’t taste it.’ And I say ‘Because you have to eat.’ So, because he doesn’t even want to eat now, that’s the problem I’m dealing with now. But now, they’re threatening him with-- if you lose any more weight, you’re going to get a feeding tube. So now it’s like ‘Give me potatoes!’ (laughs).”—Janice
	“I’m starting to feel defeated. Because I’m like if you lose any more weight, you know, and I’m so afraid for this, it’s like—he is doing the best he can. He’s eating. He is eating. He’s just not eating as much. He went from eating a whole bowl of oatmeal, to a half a bowl, to now it’s a couple of bites. I’ll just drink an Ensure and I’ll be alright and I’m watching this. And in his mind, he’s eating a lot, he’s doing great. And I’m like “Really?” (laughs)…you’re losing weight. I’m worried. I’m starting to worry. I just don’t know what else to do.”—Janice
	“We don’t have normal appetite because he doesn’t (either).”—Douglas
	“The chemo gives him the cold. You can’t touch cold, so he has to drink room temp, and if not, he can feel it in his throat. I don’t know how to describe it. When I had it, it felt like you’re swallowing glass. His hasn’t come to that yet, but his hands and feet and that, when he touches cold, it affects him, and that’s from the chemo, and we’re expecting that.”—Lenora
	“His taste has completely left, he has no taste. Um, extremely dry mouth. A lot of pain swallowing, the skin is starting to turn colors. He has lost a lot of weight I’d say. He started out at 210, he’s 197 now. And the doctor is like “No! Beef him up and…” Let’s see what else…Irritable (laughs), quick.” “Oh—and he’s got the ulcers, he has the open ulcers in the mouth. They warned us…but it’s nothing like actually going through it.”—Janice
	“Oh, yes, the vomiting, nauseousness, loss of hair, fatigue. Of course you’re gonna have fatigue with cancer, and yeah. We expected them. I went through it three years ago, so I kinda knew what he was gonna do, and he did too, because he was my caregiver, so we were kind of blessed that way (laughs), you know?”—Lenora
	“He has side effects…Mostly cramps, fatigue, some pain—he don’t sleep very well. He’s depressed…I think my role is to support him…Try to help him hang on.”—Douglas
Complementary and alternative medicine in conflict with traditional oncology protocols	“Yeah, they offered some—you know, the first thing is—well, first off, if I back up, she had some constipation, because of the pain they did give her a little oxycodone, very small amounts, which constipated her. So, they gave her Colace. So, their basic thing is to drug it and then the diarrhea, they were saying the Imodium. But we did talk to the naturopath there who we really respect and she really respects, I mean ’cause she—they talk their own lingo, so they’re—so the naturopath has been very helpful because her thing is food and things.”—Michael
	“For the bone, she was taking (stutters) some joint support, some (pause) different herbs and things like that and it’s a whole—it’s about 30 some herbs and stuff, but she was taking a lot of that. She was taking some stuff to build up the blood. She was taking some stuff to, like—guava and some other things that would help attack the cancer. So she was doing a lot of herbal stuff to actually attack and treat the cancer that was non- “medicinal” and this was prior to getting (stutters) the herbs.”—Michael
	“So—and the truth is, as far as pain goes, when we got to the hospital in December, before—when we just—and the doctors kind of said—we didn’t meet the naturopath, that was the only one we didn’t meet, unfortunately, and they told her to stop all the herbs, she walked in, she couldn’t walk out. The pain had increased. When she started just saying screw it, I’m gonna start taking the herbs actually decreased her pain, and we’re talking like 8 to 10 down to like a 3 to 4 pain.”—Michael
	“I just have to force it on him, and remind him. I tell him “it’s breakfast”—because we’re on a schedule—“It’s lunch, it’s dinner.” I became more forceful with him. Before I was—you do what you gotta do. But now I’m the one that’s more forceful. And, I try to make things a bit more seasoned, than I would normally do, because hopefully he’d be able to taste it (laughs). And then he’s throwing it out because he doesn’t want to eat it all. So now I’m starting to feel bad about that. Now you’re wasting food. I don’t know…I just keep asking for tips and hints on the internet, the doctors, somebody give me some tips. Because I don’t know what else to do, because he’s not eating.”—Janice
	“I’m thinking the bone broth and then protein shakes rather than that Ensure refrigerator. [Laughs] Yeah. We’re both not into the Ensure, that’s for sure. Although she was because part of her problem before she was diagnosed was she was having trouble eating. So, she was eating—she was downing Slimfast shakes because she was forcing herself to eat something. And you know, I’m like, let me make these shakes for you. They’re good, you know. It’s plant-based protein and coconut oil, fruits and greens. So, she has had those with me and she is open to that. I said, ‘Before you drink anything like that other stuff, please let me just do this with you.’ [Laughs]”—Beth
Be warned, but you’re on your own to figure this out	“They just recommended that kids like Doritos because they have a strong taste, so that, that (laughs) was like the only thing that kids would eat cause it, ah, ah…I don’t know, it has a strong taste so that, that would make them taste a little bit, but ah…There was, there was nothing you could do about it, about not having taste buds…” Interviewer: “Did you try the Doritos?” “I think I did (laughs)…” Interviewer: “Could you taste them?” “No.”—Damien
	“I would try to eat something different at every day just to see if something ah, made sense, if something changed. Ah, there was this different taste I could catch from something in the…I tried, I tried, but it didn’t happen. Nothing would taste…like anything.”—Damien
	“I’m thinking the bone broth and then protein shakes rather than that Ensure refrigerator. [Laughs] Yeah. We’re both not into the Ensure, that’s for sure. Although she was because part of her problem before she was diagnosed was she was having trouble eating. So, she was eating—she was downing Slimfast shakes because she was forcing herself to eat something. And you know, I’m like, let me make these shakes for you. They’re good, you know. It’s plant-based protein and coconut oil, fruits and greens. So, she has had those with me and she is open to that. I said, ‘Before you drink anything like that other stuff, please let me just do this with you.’ [Laughs]”—Beth
	“I just have to force it on him, and remind him. I tell him “it’s breakfast”—because we’re on a schedule—“It’s lunch, it’s dinner.” I became more forceful with him. Before I was—you do what you gotta do. But now I’m the one that’s more forceful. And, I try to make things a bit more seasoned, than I would normally do, because hopefully he’d be able to taste it (laughs). And then he’s throwing it out because he doesn’t want to eat it all. So now I’m starting to feel bad about that. Now you’re wasting food. I don’t know…I just keep asking for tips and hints on the internet, the doctors, somebody give me some tips. Because I don’t know what else to do, because he’s not eating.”—Janice
	“So—and the truth is, as far as pain goes, when we got to the hospital in December, before—when we just—and the doctors kind of said—we didn’t meet the naturopath, that was the only one we didn’t meet, unfortunately, and they told her to stop all the herbs, she walked in, she couldn’t walk out. The pain had increased. When she started just saying screw it, I’m gonna start taking the herbs actually decreased her pain, and we’re talking like 8 to 10 down to like a 3 to 4 pain.”—Michael
	“For the bone, she was taking (stutters) some joint support, some (pause) different herbs and things like that and it’s a whole—it’s about 30 some herbs and stuff, but she was taking a lot of that. She was taking some stuff to build up the blood. She was taking some stuff to, like—guava and some other things that would help attack the cancer. So she was doing a lot of herbal stuff to actually attack and treat the cancer that was non—“medicinal” and this was prior to getting (stutters) the herbs.”—Michael
	“But her pain is much more intense now in the evening and in the middle of the night and stuff, and it wakes her up and, you know, sometimes she is like screaming in pain and stuff like that. And so I think activity keeps you off of the pain. When you’re inactive and you’re not doing anything then you can dwell on it. And I also think you dwell on it, you know, in a negative way because it hurts, it hurts, it hurts, and you can’t distract yourself from it.”—Michael
	“It started out with everything tasted like metal for a couple weeks. And now, since last week, he has zero taste (laughs). Unless it’s super strong, like if he eats something that has citric acid, he’ll taste a twinge of it or if it’s super spicy. Other than that, he has zero taste. So, I try my best—‘You got to try to imagine it, try to imagine it. Eat with your eyes.’ But that doesn’t work, of course. So I just say—‘Just let it go down. When your stomach feels full, stop.’ (Laughs). But it’s like—trying to get him to want to eat. And he says ‘Well what’s the point? I can’t taste it.’ And I say ‘Because you have to eat.’ So, because he doesn’t even want to eat now, that’s the problem I’m dealing with now. But now, they’re threatening him with—if you lose any more weight, you’re going to get a feeding tube. So now it’s like ‘Give me potatoes!’ (laughs).”—Janice
	“Well the thing they did not they did not…they did not clue me on ahead of time was that the low dose radiation would wipe out my taste buds. For, for a significant period of time, probably, a good month or 6 weeks. That’s tough! It’s tough to lose your ability to taste food. You know, I mean pile that on top of questionable appetite you know…That I wasn’t expecting and that was probably, through all of this, I’d have to say, through all the fatigue, fatigue and everything I can handle it, but the inability to taste food properly, and then when the buds came back. It came back in waves like salt, you know so everything tasted salty you know, or bitter, or lack of sweet sense, and so you’d eat something that’s supposed to be nice and sweet and yummy and you know it’s just ‘psst’. I’ll tell you it’s a really, really, really tough time.—Roy

**Table 5:** Key Considerations and recommendations to improve person-centered care.

Through-Lines	Key Considerations and Recommendations
Caregiver empathy and sharing experience of treatment	Caregivers share the psychosocial and emotional toll of treatment side effects and are at high risk for their own negative physical, psychosocial and emotional consequences. Considering their high sense of responsibility in alleviating side effects, caregivers should be engaged by healthcare professionals during patient care. Developing and testing interventions that improve caregivers’ nutrition and long-term health and increase their capacity to provide high-quality care is a top priority. Future research is needed to develop nutrition and culinary dyadal interventions that harness the influential role that each member of the dyad holds, while simultaneously being responsive to their unique needs.
Complementary and alternative medicine in conflict with traditional oncology protocols	Open communication among patients, caregivers and cancer care providers is prerequisite to person-centered care and should be inclusive of patient preferences and their use and interest in CAM. Integrative oncology, or the use of complementary and alternative strategies alongside conventional therapies, should be promoted as a key component of person-centered care.
Be warned, but you’re on your own to figure this out	Providers should be transparent about anticipated side-effects and their management. Providers should share experiences of patients and caregivers such as these to both be transparent about the severity of side-effects and to reassure the patient or family that they are not alone or unique in their struggle.

## Data Availability

The data generated during and/or analyzed during the current study are available upon request.

## Supplementary Materials

The following are available online at https://www.mdpi.com/article/10.3390/nu14020356/s1, Table S1: Sample Participant Descriptions.
