# Supplementary material for: When Eating Becomes Torturous: Understanding Nutrition-Related Cancer Treatment Side Effects among Individuals with Cancer and Their Caregivers
Source: Nutrients. 2022 Jan 14;14(2):356. doi: 10.3390/nu14020356 (PMC8781744; doi:10.3390/nu14020356)
Supplement: Supplementary file 1 [file nutrients-14-00356-s001.zip › nutrients-1551570-supplementary.pdf]

Supplementary Table S1. Sample Participant Descriptions

Participant	Role	Age group	Gender	Cancer type	Days/wk caregiving	Hour/day caregiving
Damien	Patient	Young adult	Male	Hematologic		
Douglas	Caregiver	Adult	Male		4 day	6 hrs
John	Patient	Older adult	Male	Other solid		
Janice	Caregiver	Adult	Female		7 days	10 hrs
Beth	Caregiver	Older adult	Female		7 days	9 hrs
Susan	Patient	Older adult	Female	Hematologic		
Roy	Patient	Older adult	Male	Hematologic		
Alfred	Patient	Older adult	Male	Other solid		
Carter	Patient	Young adult	Male	Other solid		
Michael	Caregiver	Older adult	Male		7 days	6 hrs
Lenora	Caregiver	Older adult	Female		7 days	8 hrs
Henry	Patient	Older adult	Male	Other solid		

This table provides a basic description of a sample of individuals who participated in the in-depth interviews. The names provided are pseudo-names and are not linked to the participant in any way.

Age group: Young adult: 18 to 34 years; Adult: 35-54 years; Older adult: 55 years and older.
